# Enhancing antitumor immunity: the role of immune checkpoint inhibitors, anti-angiogenic therapy, and macrophage reprogramming

**DOI:** 10.3389/fonc.2025.1526407

**Published:** 2025-04-07

**Authors:** Chong Zhang, Hua Wang, Xinying Li, Yuxin Jiang, Guoping Sun, Hanqing Yu

**Affiliations:** ^1^ Department of Oncology, The First Affiliated Hospital of Anhui Medical University, Hefei, China; ^2^ Inflammation and Immune Mediated Diseases Laboratory of Anhui Province, Anhui Medical University, Hefei, China; ^3^ Department of Nephrology, The First Affiliated Hospital of Anhui Medical University, Hefei, China

**Keywords:** ICIS, angiogenesis, macrophage, combination therapy, biomarkers

## Abstract

Cancer treatment has long been hindered by the complexity of the tumor microenvironment (TME) and the mechanisms that tumors employ to evade immune detection. Recently, the combination of immune checkpoint inhibitors (ICIs) and anti-angiogenic therapies has emerged as a promising approach to improve cancer treatment outcomes. This review delves into the role of immunostimulatory molecules and ICIs in enhancing anti-tumor immunity, while also discussing the therapeutic potential of anti-angiogenic strategies in cancer. In particular, we highlight the critical role of endoplasmic reticulum (ER) stress in angiogenesis. Moreover, we explore the potential of macrophage reprogramming to bolster anti-tumor immunity, with a focus on restoring macrophage phagocytic function, modulating hypoxic tumor environments, and targeting cytokines and chemokines that shape immune responses. By examining the underlying mechanisms of combining ICIs with anti-angiogenic therapies, we also review recent clinical trials and discuss the potential of biomarkers to guide and predict treatment efficacy.

## 1 Introduction

Tumors are characterized by fourteen core hallmarks, including avoiding immune destruction, inducing and accessing vasculature, evading growth suppressors, and unlocking phenotypic plasticity, among others (1). Therefore, remodeling the tumor microenvironment to support immunity and target the vascular system is a key strategy in cancer treatment.

Currently, the use of immune checkpoint inhibitors and therapies targeting macrophages within the immune microenvironment are important components for enhancing anti-tumor immunity. The use of immune checkpoint inhibitors has significantly advanced the field of tumor immunotherapy. In recent years, various PD-1(Programmed cell death protein 1)/PD-L1(Programmed death-ligand 1) antibodies, including pembrolizumab, nivolumab, avelumab, durvalumab, and atezolizumab, have gained FDA(Food and Drug Administration) approval for treating melanoma, head and neck cancer, lymphoma, urothelial cancer, breast cancer, lung cancer, and renal cell carcinoma (2, 3). In a phase 2 clinical trial of metronomic chemotherapy combined with PD-1 inhibitors for the treatment of breast cancer, the group receiving metronomic cyclophosphamide, capecitabine, and metronomic vinorelbine combined with toripalimab showed a higher disease control rate (DCR) of 69.7% and a longer median progression-free survival (PFS) of 6.6 months compared to the other groups (4). We wonder if metronomic use of anti-angiogenic agents or immune checkpoint inhibitors could be a treatment option for patients, which requires further research.

Tumors require the rapid development of new vascular networks to sustain their growth. However, the immature nature of tumor vessels not only facilitates the creation of a tumor-inhibitory microenvironment but also causes harm to the host organism (5), therefore, this has led to the development of anti-angiogenic therapies. Preclinical animal model studies have shown that exposure to anti-angiogenic drugs leads to transient vascular changes over time, characterized by reduced vascular density, increased pericyte coverage, and enhanced perfusion. This phenomenon is referred to as the “vascular normalization window.” During this window, tumor hypoxia levels decrease, and increased perfusion can enhance the efficacy of treatments such as immune checkpoint inhibitors (ICIs) or tumor-targeted vaccines. However, the optimal timing and duration of the normalization window vary depending on the tumor type and treatment dosage. Can we identify specific biomarkers to determine the best timing for intervention? Furthermore, some *in vivo* studies suggest that administering below-standard doses may induce vascular normalization and beneficial immunomodulation. However, data on low-dose treatments in human clinical applications are limited (6, 7).

## 2 Immune stimulatory molecules and immune checkpoint inhibitors

**Figure 1:**
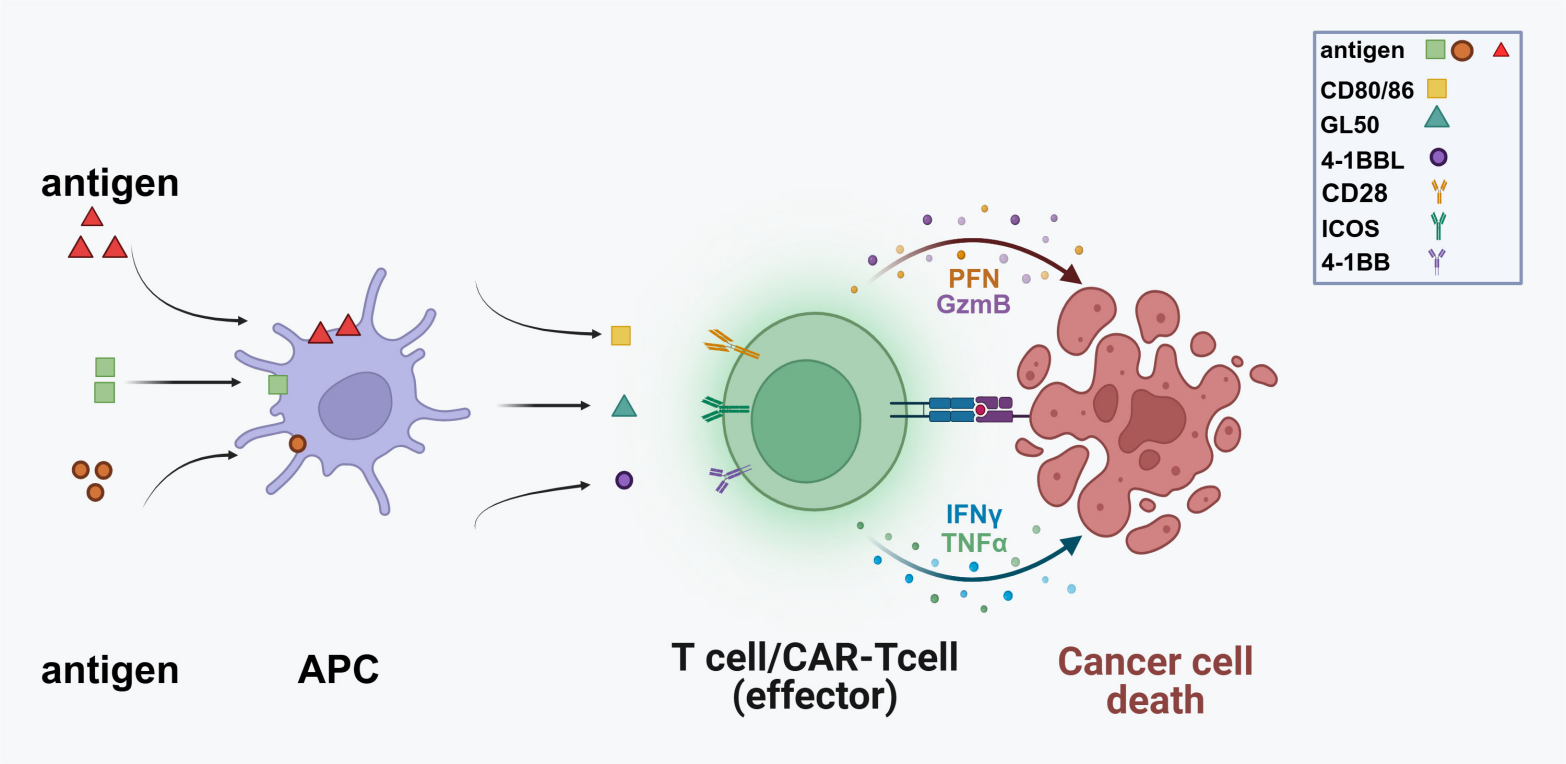
Antigen-presenting cells (APCs) display antigens derived from pathogens or malignant cells on their surfaces, facilitating recognition by T cells. Following this recognition, T cells become activated and differentiate into effector cells, including CAR-T cells specifically designed to target cancer antigens. Co-stimulatory molecules such as CD80/86, GL50, 4-1BBL, and ICOS further enhance this activation process. Subsequently, effector T cells migrate towards cancer cells, where CAR-T cells engage with antigens and release cytotoxic molecules, leading to apoptosis. (PFN, Perforin; GzmB, Granzyme B; TNFα, Tumor Necrosis Factor-alpha).

Immune checkpoint molecules include both costimulatory and co-inhibitory molecules. Costimulatory molecules, such as CD28, 4-1BB, and ICOS, are essential for activating tumor-specific T cells, leading to their proliferation and activation for effective tumor clearance. Studies have shown that CAR-T cells co-stimulated with 4-1BB or ICOS exhibit prolonged persistence in xenograft models, allowing for the elimination of recurrent and refractory tumors (8) (**Figure 1**). Immune stimulatory molecules play a crucial role in regulating immune responses; however, their excessive activation can lead to various diseases, such as systemic lupus erythematosus and rheumatoid arthritis. Therefore, it is essential to carefully regulate the intensity and type of immune stimulation during treatment to prevent the occurrence of these adverse reactions.

Immune checkpoint inhibitors (ICIs), such as CTLA-4, PD-1, and PD-L1, inhibit the proliferation of tumor-specific T cells and induce their depletion. This impairs the body’s ability to mount a robust anti-tumor immune response, leading to tumor escape (9–12). In 2010, a breakthrough occurred during the clinical trials of the CTLA-4 monoclonal antibody ipilimumab for treating unresectable metastatic melanoma. These studies demonstrated a marked improvement in patients’ overall survival rates. Subsequently, in 2011, the United States FDA approved ipilimumab for treating advanced melanoma, setting a precedent for immune checkpoint blockade in cancer treatment (13).

Immune checkpoint molecules maintain immune balance through the intricate regulation of activation and inhibition. This process is highly complex and delicate, requiring tight control to avoid negatively impacting the body’s immune system.

### 2.1 PD1

PD-1 can be induced and expressed in various immune cells, including B cells, CD4+ T cells, CD8+ T cells, NK cells, and activated monocytes (14).

This receptor interacts with its ligand PD-L1, leading to the recruitment of the tyrosine phosphatase SHP2. Upon binding, PD-1 facilitates the dephosphorylation of nearby effector proteins, inhibiting T cell proliferation, causing metabolic reprogramming, and reducing cytokine secretion. These mechanisms collectively suppress T cell function and activation (15, 16).

**Figure 2:**
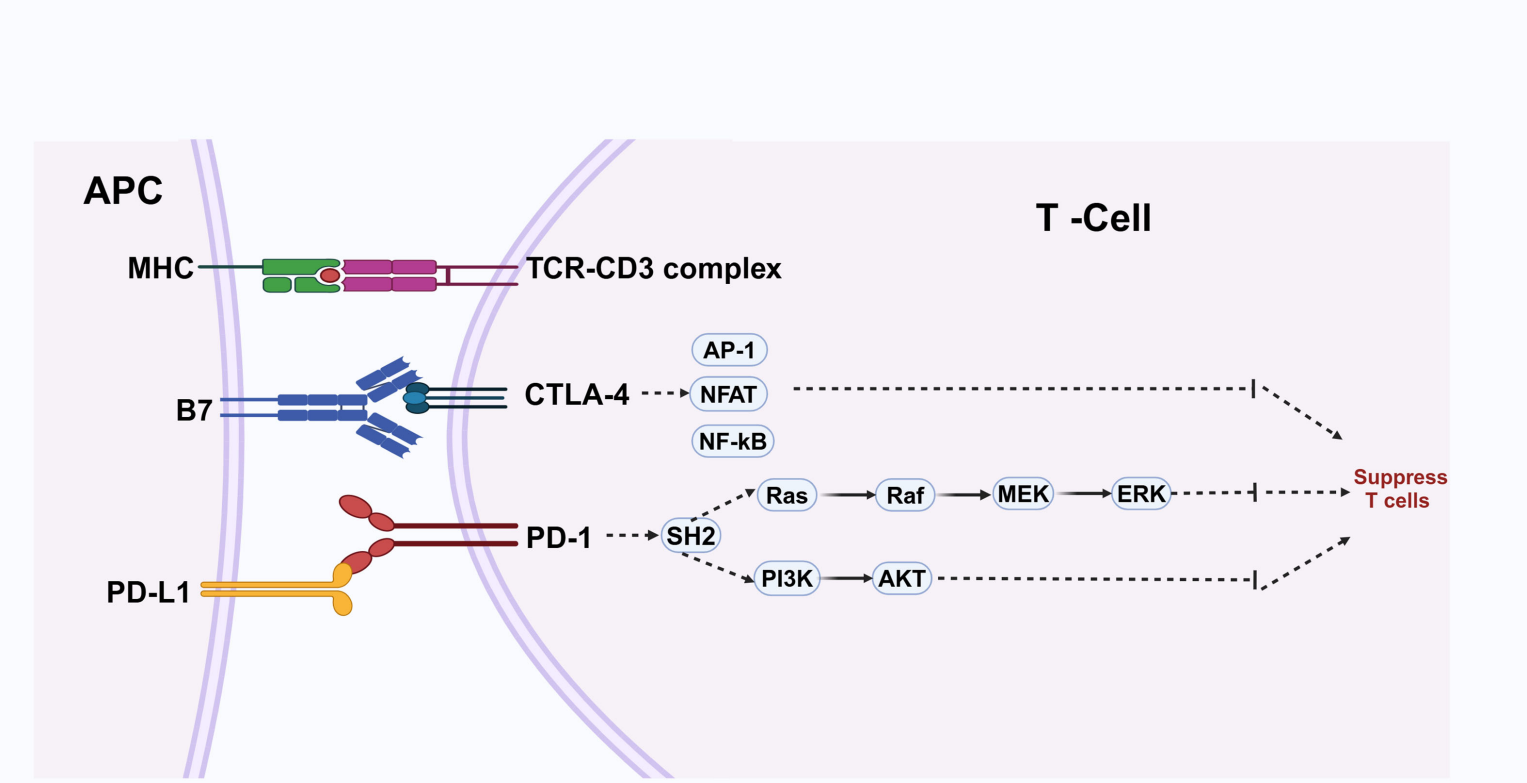
Antigen-presenting cells (APCs) display antigens through major histocompatibility complex (MHC) molecules to the T-cell receptor (TCR)-CD3 complex, activating T cells in conjunction with co-stimulatory signals from B7 molecules interacting with CTLA-4. This activation process engages transcription factors such as AP-1, NFAT, and NF-κB. Conversely, PD-L1 expressed on APCs binds to PD-1 on T cells, initiating pathways that inhibit T cell activity and facilitate immune evasion by cancer cells. Maintaining a balance between these signals is essential for immune homeostasis.

Immune checkpoint suppressor molecules play a vital role in down-regulating the intensity of immune responses and contributing to peripheral tolerance, thereby maintaining immune homeostasis. However, tumor cells can up-regulate immune checkpoint signals, such as PD-1/PD-L1, enabling them to evade the immune system (17) (**Figure 2**).

In some patients undergoing PD-1 immunotherapy, there is a risk of rapid cancer progression, known as hyper-progressive disease (HPD). Interestingly, studies suggest that eliminating e-Treg cells from tumor tissue can effectively treat and prevent HPD during PD-1 treatment (18).

### 2.2 PD-L1

PD-L1 (CD274) can be expressed on various immune cells, including B cells, T cells, dendritic cells, and macrophages. This broad expression of PD-L1 highlights its extensive role in immune regulation across different cell types (19, 20).

PD-1, a surface receptor, binds to two ligands: PD-L1 and PD-L2. Several studies have demonstrated that the interaction between PD-1 and its ligands is crucial for controlling the induction and maintenance of peripheral T cell tolerance. This interaction regulates immune responses and prevents excessive T cell activation, thereby maintaining immune homeostasis (21).

Anti-PD-1/PD-L1 therapy has been shown to upregulate the Ras-Raf-MEK-ERK and PI3K-AKT signaling pathways in immune cells by blocking the PD-1/PD-L1 axis. This mechanism can lead to the recovery of T cells from a dysfunctional state and enhance their tumor-killing activity. By upregulating these critical signaling pathways, anti-PD-1/PD-L1 therapy can amplify the immune response and facilitate effective tumor clearance (22).

The role of PD-L1 in tumor immunity is particularly important in the context of treatment. Notably, there is a significant disparity in the effectiveness of PD-1 or PD-L1 blocking therapy between patients who are PD-L1 positive and those who are PD-L1 negative (23) (**Figure 2**).

### 2.3 CTLA-4

CTLA-4 is predominantly found within the intracellular vesicles of FoxP3+ regulatory T cells (Tregs) and activated conventional T cells (24). CTLA-4 and CD28 are homologous receptors expressed by both CD4+ and CD8+ T cells, allowing them to bind to the same ligands, CD80 and CD86, with CTLA-4 exhibiting a higher affinity. Structurally, CTLA-4 is present on the surface of immunosuppressive Tregs, making it a potential target for applications in immunosuppression (25, 26). CD28 and CTLA-4 have contrasting roles in T cell activation: CD28 provides a stimulatory signal, while CTLA-4 delivers an inhibitory signal, thereby stabilizing T cell immune regulation (27). CTLA-4 inhibits T cell activity by influencing various transcription factors involved in T cell activation, proliferation, and cytokine production, including NF-kB, NFAT, and AP-1 (28). Consequently, the CTLA-4 pathway is a critical component of immune-based cancer therapies and treatments for autoimmune diseases and CTLA-4 deficiency (29).

A recent study revealed that the absence of the co-inhibitory receptor CTLA-4 might benefit T cell function in leukemia patients who experienced treatment failure with CAR-T cell therapy. Additionally, CTLA-4 loss can enhance CAR-T cell proliferation and improve their anti-tumor efficacy (30). However, caution is necessary when approaching anti-CTLA-4 therapy. One study indicated that melanoma tumors treated with anti-CTLA-4 therapy exhibit significant changes, including an elevated tumor mutation burden, increased inflammatory characteristics, and alterations in cell cycle processes, compared to untreated tumors (31) (**Figure 2**).

### 2.4 Emerging immune checkpoint targets beyond conventional pathways

In addition to PD-1, PD-L1, and CTLA-4, there are several other promising immune checkpoint inhibitor (ICI) drug targets. For instance, CD47 is a transmembrane protein that is widely expressed on a variety of cell surfaces. It binds to the signaling regulatory protein α (SIRPα) on macrophages, transmitting inhibitory signals that prevent macrophages from phagocytosing CD47-expressing cancer cells (32). CD47 inhibitors work by blocking the CD47-SIRPα signaling axis, effectively releasing tumor cells from immune evasion by macrophages, activating macrophage phagocytic function, and thereby enhancing the body’s anti-tumor immune response. Currently, multiple drugs targeting CD47 are in various stages of clinical research worldwide, including monoclonal antibodies, bispecific antibodies, and fusion proteins (33).

LAG-3 (lymphocyte activation gene 3) is a co-inhibitory molecule expressed on activated T cells, B cells, and natural killer cells. LAG-3 binds to major histocompatibility complex class II (MHC-II) molecules, transmitting inhibitory signals that lead to T cell functional exhaustion. LAG-3 inhibitors can block this signaling pathway, restoring T cell anti-tumor activity. Various LAG-3 inhibitors are currently in clinical trial stages (34).

TIGIT (T cell immunoglobulin and ITIM domain) is another co-inhibitory molecule expressed on T cells and natural killer cells. TIGIT inhibits the activation and proliferation of T cells and natural killer cells by binding to CD155. TIGIT inhibitors can disrupt this signaling pathway, thereby enhancing the anti-tumor activity of immune cells. Several TIGIT inhibitors are also undergoing clinical trials (35).

Future research will increasingly focus on combination treatment strategies involving immune checkpoint inhibitors (ICIs). For example, the combined application of PD-1/PD-L1 inhibitors and CTLA-4 inhibitors has been confirmed in some clinical trials to enhance anti-tumor immune responses through synergistic effects. Notably, molecules such as LAG-3, TIM-3, and TIGIT are emerging as new generation immune checkpoint receptors with clinical applicability. A deeper understanding of the unique biological processes regulated by these receptors in immune cells and tissues (including T cell exhaustion dynamics, immune synapse formation, and metabolic reprogramming) will provide crucial guidance for developing antagonists or agonists targeting these receptors. The mechanistic research will directly influence the optimization of clinical translation pathways and the formulation of individualized treatment strategies.

## 3 Angiogenesis

### 3.1 Vascular endothelial growth factor

One contributing factor to the lack of normalization in tumor vessels is the abnormal secretion of growth factors by tumor and stromal cells, with VEGF (Vascular endothelial growth factor) playing a crucial role (36). The angiogenic signal of VEGF is primarily mediated by its receptor, VEGFR2. The VEGF-VEGFR2 signaling pathway activates downstream PLCγ-PKC-Raf-MAPK and Grb2/Gab1-MAPK/PI3K-Akt pathways, leading to the secretion of von Willebrand factor (vWF) and promoting endothelial cell (EC) proliferation and migration. Additionally, VEGF-VEGFR2 enhances vascular permeability through the activation of VEGFR2-TSAd-Src-cadherin and PI3K-Akt-eNOS signaling pathways (37, 38). VEGF also suppresses the phosphorylation and subsequent degradation of IκB in immature dendritic cells (DCs), thereby inhibiting the activation of NF-κB. The administration of a VEGF inhibitor, such as bevacizumab, has been shown to normalize the vascular system, reduce T-regs, and promote the maturation of DCs (39).

VEGF not only stimulates tumor angiogenesis but also plays a role in creating an immunosuppressive microenvironment. The presence of leaky neovascularization and inadequate pericyte coverage results in elevated interstitial fluid pressure within the tumor, making T cell infiltration challenging due to the significant pressure difference. Additionally, neovascularization often lacks adhesion molecules like vascular cell adhesion molecule-1 (VCAM-1), further hampering T cell extravasation (40). This inadequate neovascularization cannot compensate for the heightened oxygen consumption caused by tumors, leading to direct damage to tumor-infiltrating lymphocytes (TILs). The resulting hypoxia up-regulates anti-tumor immunosuppressive signals, including PD-L1, IL-6, and IL-10 (41). Excessive angiogenesis also increases the abundance of lymphocytes that promote tumor growth (42).

Due to irregularities in the tumor’s blood vessels, hypoxia-induced upregulation of L-22 and L-28 attracts Treg cells to the tumor microenvironment (43). VEGF-A and IL-10 can induce the expression of FasL/CD95L in vascular endothelial cells of solid tumors, allowing FasL to cause death in effector CD8+ T cells rather than Treg cells, which have high levels of cFLIP. Inhibition of VEGF through angiogenic inhibitors has been shown to decrease the expression of FasL in tumor endothelial cells (44).

In summary, angiogenesis leads to the accumulation of immunosuppressive cells within the tumor microenvironment while reducing the presence of anti-tumor immune cells. This process ultimately facilitates tumor development and progression (45).

A study led to the development of a chimeric peptide, OGS, that targets both PD-L1 and VEGFR2. OGS exhibits a high affinity for both human and mouse PD-L1, effectively blocking the interaction between PD-1 and PD-L1. When combined with a serum albumin-binding peptide, DSP, to form DSPOGS, this chimeric peptide significantly enhances the infiltration of CD8+ T cells, leading to effective anti-tumor effects when used alone or in combination with radiotherapy (46).

Combination therapy involving anti-angiogenesis and anti-PD-L1 has been shown to effectively stimulate tumor immunity by facilitating the formation of high endothelial venules (HEVs) (47). Combined treatment with anti-VEGFR2 and anti-PD-L1 antibodies has been effective in inducing HEVs in both breast cancer and pancreatic neuroendocrine tumors. HEVs, being specialized post-capillary venules, play a crucial role in facilitating the entry of lymphocytes from the bloodstream into secondary lymphoid organs. The activation of the lymphotoxin β receptor (LTβR) signaling pathway by these HEVs further enhances lymphocyte infiltration and activity (47). The induction of intra-tumor HEVs through combined therapy may exhibit similar functional characteristics to those of normal HEVs found in lymph nodes. These induced HEVs have the potential to enhance T cell infiltration within the tumor, consequently facilitating the effectiveness of anti-angiogenesis and anti-PD-L1 therapy for the tumor. This, in turn, can contribute to the improvement of therapeutic outcomes (47).

**Figure 3:**
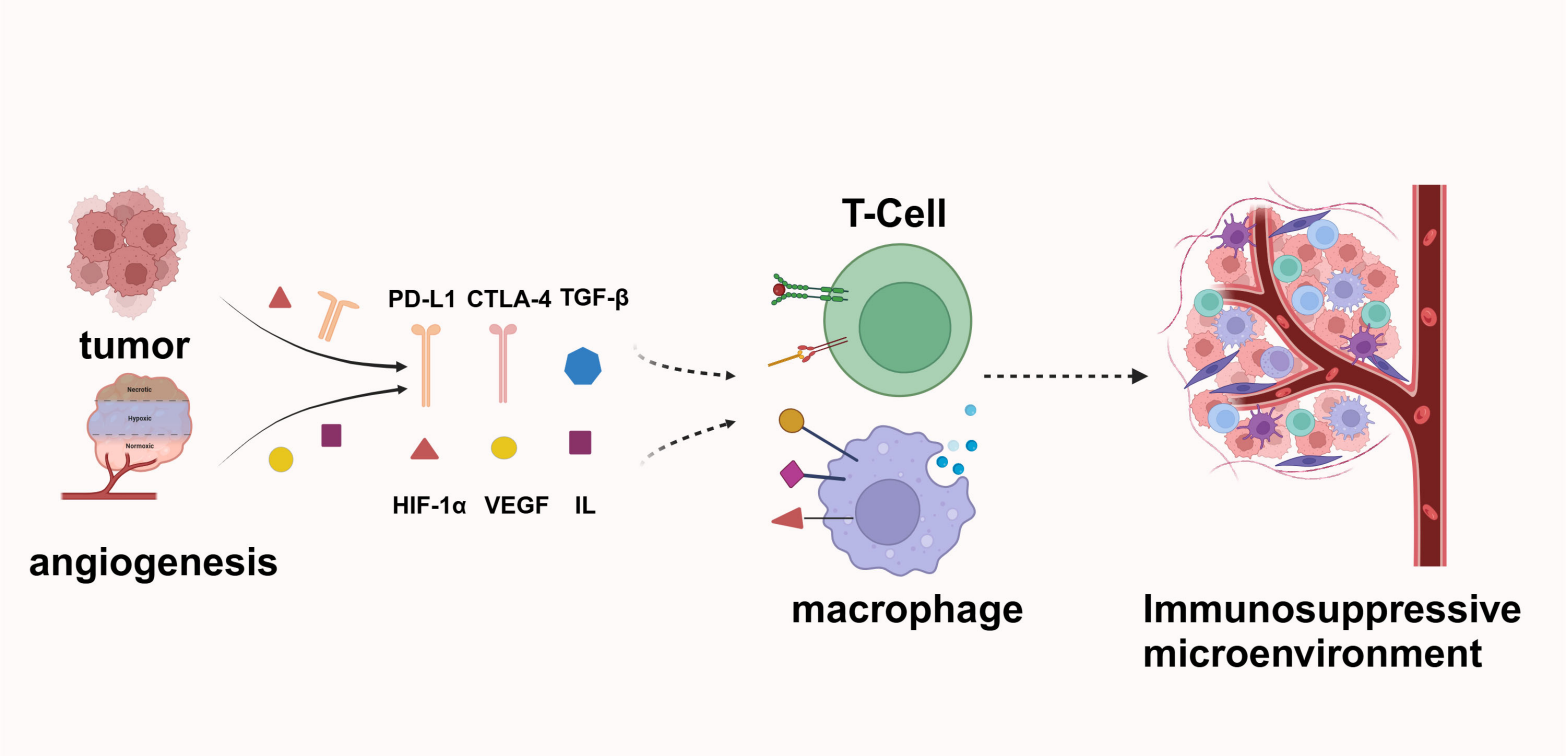
Tumor cells secrete factors such as PD-L1, CTLA-4, and TGF-β to inhibit T-cell activity and evade immune surveillance. The tumor microenvironment, characterized by the presence of Hypoxia-Inducible Factor 1-alpha (HIF-1α), VEGF, and various interleukins, facilitates angiogenesis and supports tumor growth. Additionally, tumor-derived signals polarize macrophages, thereby creating an immunosuppressive environment that further enhances tumor immune evasion.

Angiogenesis plays a significant role in immune evasion. Blood vessels are crucial for enabling circulating immune cells to infiltrate and eliminate tumors. However, the aberrant vascular architecture in tumors creates an immune barrier and disrupts normal blood flow and oxygenation. This abnormal neovascularization fails to meet the heightened oxygen demands of tumors, resulting in tumor hypoxia. Hypoxia directly impairs the function of tumor-infiltrating lymphocytes (TILs) and upregulates immunosuppressive signals such as PD-L1, TGF-β, IL-6, and IL-10 within the tumor microenvironment. Additionally, hypoxia promotes the polarization of tumor-associated macrophages (TAMs) towards an M2-like phenotype (48–50) (**Figure 3**).

Angiogenic factors can stimulate tumor endothelial cells to create a barrier that obstructs immune cell infiltration. Endothelial cells can express immune inhibitory molecules that suppress leukocyte function, acting as immune checkpoints, and can also induce apoptosis in immune cells (51).

Thus, anti-vascular drugs are of significant importance. Anti-angiogenic drugs targeting VEGF and VEGFR are extensively used in clinical cancer treatments. These drugs also have the potential to enhance immune responses (52, 53).

### 3.2 Anti-angiogenic therapy in cancer treatment

Tumor vascular normalization and immune microenvironment reprogramming exhibit a mutually regulated relationship, making anti-vascular therapy pivotal in tumor treatment (54). Angiogenesis inhibitors have the potential to reshape the tumor immune microenvironment in several ways. Firstly, anti-tumor angiogenesis can alter the proportion of anti-tumor and pro-tumor immune cells within the microenvironment. Additionally, it can remodel the function of cytotoxic T lymphocytes (CTLs), ultimately creating a microenvironment more conducive to promoting tumor immunity (55). Furthermore, anti-angiogenic therapy can activate immune response-derived factors, including IFN-γ and TGF-β, facilitating vascular normalization and regression within the tumor microenvironment (56). Moreover, anti-angiogenesis can selectively inhibit the expression of immune checkpoint molecules, such as PD-1 and CTLA-4, on intra-tumoral CD8+ T cells. This suppression alleviates the inhibitory signals that hinder the antitumor immune response, thereby enhancing the efficacy of immunotherapy (57).

Several studies have demonstrated diverse effects of anti-angiogenic therapy. For example, anlotinib, a novel multi-target tyrosine kinase inhibitor primarily targeting VEGFR2/3, FGFR1-4, and other receptors, showed manageable toxicity and broad-spectrum antitumor potential in patients with advanced refractory solid tumors (58). Another study aimed to investigate the antitumor activity and tolerability of apatinib, an oral small molecule anti-angiogenic inhibitor, in patients with recurrent advanced melanoma. Out of the 15 patients who received apatinib treatment, 11 achieved stable disease, resulting in a disease control rate (DCR) of 86.7%, and a median overall survival (OS) of 12.0 months. These results indicate that apatinib exhibits potential as a second-line or above-line treatment option for patients with malignant melanoma (59).

It has long been recognized that drugs inhibiting angiogenesis or disrupting established tumor vasculature can slow cancer progression. However, the standard dosing of anti-angiogenic agents in clinical applications has shown limitations. The destruction of blood vessels often leads to hypoxia, which can accelerate tumor progression. In fact, compelling evidence suggests that adding low doses of anti-angiogenic drugs to immune checkpoint inhibitors (ICIs) significantly enhances antitumor immunity.

In a mouse model of breast cancer, the combination of anti-PD-1 therapy with various doses of VEGFR2-targeted agents was evaluated. The results indicated that both conventional and low-dose anti-VEGFR2 antibody treatments normalized tumor vasculature; however, low-dose VEGFR2 blockade resulted in more robust immune cell infiltration and activation, promoting CD8 T cells to secrete osteopontin (OPN). Subsequently, OPN induced tumor cells to produce TGF-β, which upregulated PD-1 expression on immune cells. In patients with advanced triple-negative breast cancer (TNBC), the combination of low-dose VEGFR2 inhibitors and anti-PD-1 therapy demonstrated excellent tolerability and efficacy. Higher expressions of OPN and TGF-β were associated with improved therapeutic responses (60).

In another study, the effects of combining anlotinib with immune checkpoint therapy were investigated in a mouse model of breast cancer. The results indicated that effective low doses of anlotinib were sufficient to inhibit tumor growth while reducing side effects compared to high doses. Low-dose anlotinib treatment induced persistent normalization of tumor vasculature and improved the efficacy of anti-PD-1 therapy in both short-term and long-term treatment regimens. Mechanistically, the combination therapy increased the proportions of intratumoral CD4 T cells, CD8 T cells, and NK cells. These results suggest that the combination of effective low-dose anlotinib and PD-1 blockers can induce sustained antitumor effects with fewer side effects (61).

### 3.3 Endoplasmic reticulum stress and angiogenesis

When cells undergo damage from factors such as infection, inflammation, oxidative stress, or malnutrition, the endoplasmic reticulum’s (ER) function is hindered, leading to disrupted protein metabolism and potential endoplasmic reticulum stress (ERS) (62). ERS can result in the accumulation of unfolded or misfolded proteins in the ER lumen, triggering an adaptive system called the unfolded protein response (UPR), which alleviates ERS and restores protein homeostasis (63). In mammalian cells, three proteins that span the ER membrane—IRE1 (inositol requiring enzyme 1), PERK (PRKR-like endoplasmic reticulum kinase), and ATF6 (activating transcription factor 6)—mediate distinct UPR signaling pathways that play crucial roles in relieving ERS and maintaining normal cellular function (64).

**Figure 4:**
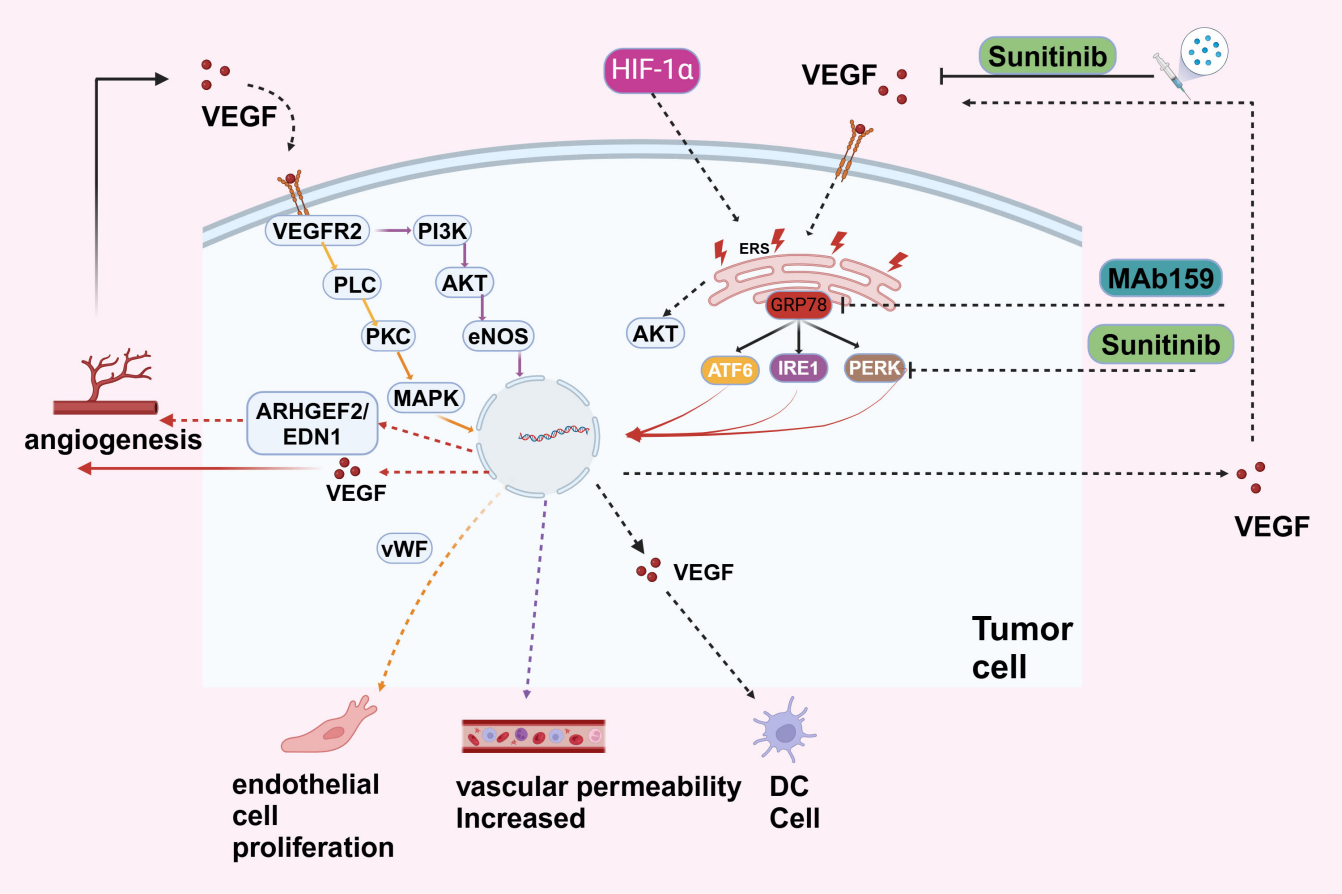
VEGF, secreted by tumor cells, binds to VEGFR2 on endothelial cells, activating pathways like PI3K-AKT (promoting cell survival and growth), PLC-PKC (stimulating nitric oxide production via eNOS), and MAPK (driving cell proliferation through ARHGEF2 and EDN1). These pathways result in endothelial cell proliferation, increased vascular permeability, and angiogenesis. Hypoxia-Inducible Factor 1-alpha (HIF-1α) upregulates VEGF under low oxygen conditions, further promoting angiogenesis and supporting tumor growth and metastasis. The endoplasmic reticulum stress response in tumor cells, involving proteins like GRP78, ATF6, IRE1, and PERK, activates pro-survival pathways, including AKT signaling, aiding tumor cell adaptation under stress. Therapeutic interventions like Sunitinib, a tyrosine kinase inhibitor blocking VEGFR2, and MAb159, targeting VEGF, inhibit angiogenesis and tumor growth.

ERS has a cross-interaction with tumor angiogenesis. The UPR can promote angiogenesis, and increased angiogenesis can enhance UPR signaling transduction (65, 66) (**Figure 4**).

Cancer cells, owing to their rapid growth, encounter survival pressures such as hypoxia and amino acid deprivation. These conditions activate factors like ATF4 and XBP1 in the UPR pathway, which can bind to the VEGF promoter and upregulate VEGF expression. The UPR pathway also stimulates the expression of other angiogenic factors such as IL-8, facilitating tumor growth and metastasis (67–69).

Angiogenesis also enhances the transmission of UPR signals. VEGF, a critical pro-angiogenic factor secreted by tumor cells, can activate ER stress sensors (PERK, IRE1, ATF6) in endothelial cells, even in the absence of ER stress and protein misfolding. This suggests that angiogenesis can influence UPR signaling pathways independently of ER stress conditions (70).

A variety of pharmacological agents designed to modulate the ER stress pathway have been developed and utilized (71). Sunitinib, a small-molecule inhibitor that targets multiple receptor tyrosine kinases, exhibits a dual mechanism of action: it inhibits the kinase activity of IRE1α, suppressing the splicing of XBP1, and impedes factors such as VEGFRs. Consequently, the anti-angiogenic effects of sunitinib are attributed to its combined inhibition of receptor expression in angiogenic cells and the ER stress pathway, illustrating a synergistic therapeutic approach (72, 73). Other therapeutic agents, such as SK2656157, an inhibitor of PERK, have demonstrated anti-angiogenic and anti-tumor properties in xenograft models derived from pancreatic adenocarcinoma (74). Additionally, MAb159, a high-affinity mouse monoclonal antibody targeting GRP78, modulates the PI3K pathway, inhibiting tumor growth and metastasis. A humanized version of MAb159 is anticipated to commence clinical trials soon, representing a promising development in targeted cancer therapy (75).

Certain molecular signaling pathways also hold promise as potential therapeutic targets. ER stress promotes angiogenesis and tumor growth by up-regulating ARHGEF2 and activating the EDN1 pathway, which contributes to angiogenesis and treatment resistance associated with ER stress in hepatocellular carcinoma (76). This pathway represents a potential novel target for anti-angiogenic treatment strategies.

Numerous drugs targeting the UPR pathway and angiogenesis have demonstrated potential as anticancer agents. Despite these promising findings, comprehensive preclinical studies and clinical trials are imperative to substantiate their safety profile and therapeutic benefits.

## 4 Application of immune checkpoint inhibitors and the immunosuppressive microenvironment leading to resistance

Immune checkpoint inhibitors (ICIs) have revolutionized cancer therapy by blocking specific immune checkpoint molecules, thereby reactivating the host immune system’s ability to target tumor cells.

In the early stages of ICI development, research primarily focused on the treatment of melanoma. In 2011, the U.S. Food and Drug Administration (FDA) approved the CTLA-4 inhibitor ipilimumab for the treatment of advanced melanoma, marking the first significant clinical breakthrough for immune checkpoint inhibitors (77). Ipilimumab enhances T cell activation and proliferation by blocking the interaction between CTLA-4 and B7 molecules on antigen-presenting cells, thereby exerting its anti-tumor effects. Clinical studies have demonstrated that patients with advanced melanoma treated with ipilimumab experienced a significant extension in overall survival, thereby opening a new chapter in the clinical application of immune checkpoint inhibitors (78).

Subsequently, PD-1 inhibitors emerged in clinical practice. In 2014, pembrolizumab and nivolumab were respectively approved by the FDA for the treatment of advanced melanoma (79). As research progressed, the scope of ICI application gradually expanded from melanoma to various other cancer types. In the field of non-small cell lung cancer (NSCLC), significant advancements have been made with PD-1/PD-L1 inhibitors. For patients with advanced NSCLC, particularly those with high expression of Programmed Death-Ligand 1 (PD-L1), treatment with PD-1/PD-L1 inhibitors alone significantly prolonged progression-free survival (PFS) and overall survival (OS) (80).For instance, pembrolizumab used as a first-line monotherapy for advanced NSCLC patients demonstrated significant improvement in OS and PFS compared to traditional chemotherapy, with a relatively low incidence of adverse effects (81).In renal cancer, nivolumab monotherapy for advanced renal cell carcinoma also exhibited efficacy, prolonging patient survival and enhancing quality of life (82).

Moreover, ICIs have achieved significant results in the treatment of bladder cancer, head and neck squamous cell carcinoma, and Hodgkin lymphoma, increasingly becoming an important treatment option for these malignancies (83–86).

Despite the remarkable clinical efficacy of immune checkpoint inhibitors, resistance remains a significant limitation to their widespread application. Primary resistance to anti-PD therapy is characterized by the initial ineffectiveness of this treatment in tumors that exhibit both PDL1 expression and T cell infiltration (87). Acquired resistance occurs when certain patients, after initially responding to PD-1/PD-L1 blocking therapy, eventually develop resistance or experience relapse. It has been observed that between a quarter and a third of patients with metastatic melanoma, who initially respond positively to PD therapy, eventually experience relapse. The mechanism of tumor resistance is highly intricate, with the establishment of an immunosuppressive microenvironment being a crucial aspect. Tumor cells create this sophisticated immunosuppressive microenvironment by exploiting the surrounding milieu or pre-existing immunosuppressive pathways (88). For instance, CTLA-4 and TGF-β are involved in this process. CTLA-4 is essential in suppressing autoimmune responses in healthy individuals, while TGF-β inhibits unnecessary inflammation and autoimmunity. Tumor cells can exploit these cytokines or molecules to promote their own growth and development (87).

### 4.1 The immunosuppressive microenvironment

The formation of the tumor inhibitory microenvironment is influenced by numerous factors, with tumor-associated macrophages (TAMs) playing a significant role. Macrophages differentiate from monocytes in tissues, acquiring functional phenotypes based on microenvironmental signals, which can lead to seemingly opposite functions (89).

Initially, macrophages were believed to be involved in the host’s anti-tumor response due to their abundance in tumors. However, it is now recognized that many TAMs contribute to tumor progression and metastasis. TAMs also remodel the tumor microenvironment by expressing proteases such as matrix metalloproteinases and cathepsin. Additionally, hypoxia and growth factors recruit TAM regulatory factors, leading to increased vascular density (90).

M2 tumor-associated macrophages play a crucial role in facilitating tumor advancement by secreting inhibitory cytokines that suppress immune support cells. This results in an immunosuppressive microenvironment conducive to tumor growth, proliferation, angiogenesis, and metastasis. A high abundance of TAMs in tumors is generally associated with poor prognosis. Therefore, targeting TAMs through cancer immunotherapy is crucial. Approaches may include depleting immunosuppressive factors within the tumor, hindering their tumor-promoting functions, or reinstating their immunostimulatory and tumor-killing properties. Such interventions have the potential to enhance anti-tumor immune responses and improve treatment outcomes (91, 92).

Additionally, chronic inflammation, angiogenesis, immune evasion, and the extracellular matrix contribute to the formation of the tumor immunosuppressive microenvironment (54, 93–95).

Different types of tumors may exhibit distinct microenvironmental characteristics and influencing factors. Therefore, further scientific research and a deeper understanding are necessary to fully comprehend the mechanisms behind the formation of the tumor microenvironment.

### 4.2 Immunosuppressive factors

Tumor cells can create immunosuppressive molecular pathways and develop adaptive resistance by stimulating the secretion of immunosuppressive factors in their surrounding environment (87).

Tumor cells secrete cytokines, such as CSF-1, which bind to macrophage surface receptors and regulate the expression of immunosuppressive genes, including IL-1, IL-8, IL-10, and CSF-1. This process increases the secretion of TGF-β and other tumor-promoting factors while inhibiting the secretion of anti-tumor factors, ultimately impacting the immune function of major cells within the tumor microenvironment and promoting tumor immune escape (96–101).

Tumor cells expressing high levels of self-markers, such as the CD24/Siglec-10 signal axis, phosphorylate ITIMs (immune receptor tyrosine-based inhibitory motifs), thereby activating downstream pathways and releasing anti-phagocytic signals. This process negatively regulates macrophage phagocytosis (102). M2 polarized cytokines, such as IL-4, TGF-β, and IL-10, can strongly induce the expression of Siglec-10. Interfering with CD24 or Siglec-10 has been shown to significantly enhance macrophage phagocytosis of CD24-positive tumors, thereby inhibiting tumor growth (103).

Bone marrow-derived suppressor cells (MDSCs) represent a subset of immature bone marrow cells with inhibitory capabilities within the tumor microenvironment. Additionally, bone marrow mesenchymal stem cells produce immunosuppressive factors that can impede both antigen-specific and non-specific T cell responses, thereby contributing to tumor invasion and stimulation of angiogenesis. Targeting these factors holds potential as a therapeutic approach (104).

Tumors can overexpress VEGF, an immunosuppressive factor that promotes tumor development and progression. Tumor-derived VEGF, when combined with tumor-associated macrophages, can induce high expression of PD-1 and CTLA-4 on the surface of CD8+ T cells, leading to resistance to PD-1 and PD-L1 therapy (57).

Hypoxia, a common characteristic of solid tumors, triggers the activation of hypoxia-inducible factor-1 (HIF-1), leading to the upregulation of VEGF within tumors and resulting in neovascularization (105). This process involves the inactivation of the tumor suppressor VHL, leading to an accumulation of HIF-1α. Increased levels of HIF-1α stimulate the transcription of genes responsible for encoding VEGF and PDGF, promoting tumor development and progression (106).

VEGF primarily exerts its effects by binding to its receptors, with VEGFR2 mediating the main angiogenic signals. The overexpression of VEGF and subsequent angiogenesis may be associated with immune suppression in cancer patients (107). A study conducted in animal models found an enhanced selective expression of VEGFR2 in tumor-associated bone marrow cells. VEGF induced an immune-suppressive phenotype in these VEGFR2+ bone marrow cells, including an upregulation of PD-L1 expression. Inhibition of VEGF reversed the immune-suppressive phenotype in VEGFR2+ bone marrow cells, promoting T cell activation and improving the efficacy of immune checkpoint blockade (108).

Certain factors that increase VEGF levels are likely to result in treatment resistance. For instance, the transcription factor FOXK2 is upregulated in anaplastic thyroid cancer (ATC), with its expression level correlated with tumor size. Research has found that FOXK2 can positively regulate the VEGF and VEGFR signaling network by inducing VEGFA transcription to promote angiogenesis. However, when VEGFR2 is blocked by targeted drugs such as Apatinib, FOXK2 can rapidly induce treatment resistance. Therefore, FOXK2 plays a crucial role in angiogenesis in ATC and resistance to VEGFR2 blockade by inducing VEGF (109).

### 4.3 T-cell dysfunction and other resistance mechanisms

CD8+ T cells that have failed to eliminate tumors express inhibitory receptors such as PD-1, LAG-3, and CTLA-4 (2). Thus, anti-PD-1/PD-L1 antibodies may only overcome a subset of the inhibitory signals in the tumor microenvironment (TME), leaving other axes of inhibition that impair T cell function (110). Dysfunction of the host immune system, such as T cell anergy and the excessive presence of regulatory T cells (Tregs), represents one of the primary mechanisms tumors employ to evade immune surveillance. Additionally, tumor-related factors, including the secretion of immunosuppressive cytokines, promotion of anti-apoptosis, and antigen deficiency, are crucial contributors to tumor immune escape (111).

The development of checkpoint inhibitors has greatly accelerated the clinical application of numerous single-agent and combination immunotherapies. However, the limited efficacy observed when these immunotherapies are used as monotherapies or in combination with checkpoint inhibitors indicates the need for new strategies to overcome resistance. Specific oncogenic mutations and broad genomic signatures, such as microsatellite instability (MSI), disrupt communication and recruitment between tumor cells and immune system cells. Future successful therapies will focus on two crucial factors: preserving T-cell homing and addressing dysfunction within the TME, and harnessing the function of mononuclear phagocytes for inflammatory remodeling within the TME (112, 113). A potential strategy to enhance resistance involves using anti-angiogenesis and immune checkpoint inhibitors.

Cancer cells can evade macrophage clearance by upregulating surface proteins referred to as “don’t eat me” signals. These include CD47, PD-L1, and beta-2 micro-globulin subunits of the major histocompatibility Class I complex (B2M). Notably, CD24 serves as an effective anti-phagocytic molecule that transmits the “don’t eat me” signal and directly shields cancer cells from macrophages expressing Siglec-10. Blocking the CD24-Siglec-10 signaling pathway through monoclonal antibody intervention has shown significant potential in enhancing the elimination of CD24+ tumors, highlighting the promising prospects of utilizing CD24 blocking as an immunotherapeutic approach. The CD47-SIRPα signaling pathway can also facilitate the evasion of macrophage clearance by transmitting a “don’t eat me” signal (114).

## 5 Targeting reprogramming macrophages to enhance antitumor immunity

Under normal conditions, macrophages are crucial in the immune microenvironment. While the spectrum of macrophage activation states is complex, it is often simplified into two categories: M1 classically activated macrophages and M2 alternatively activated macrophages (115).

**Figure 5:**
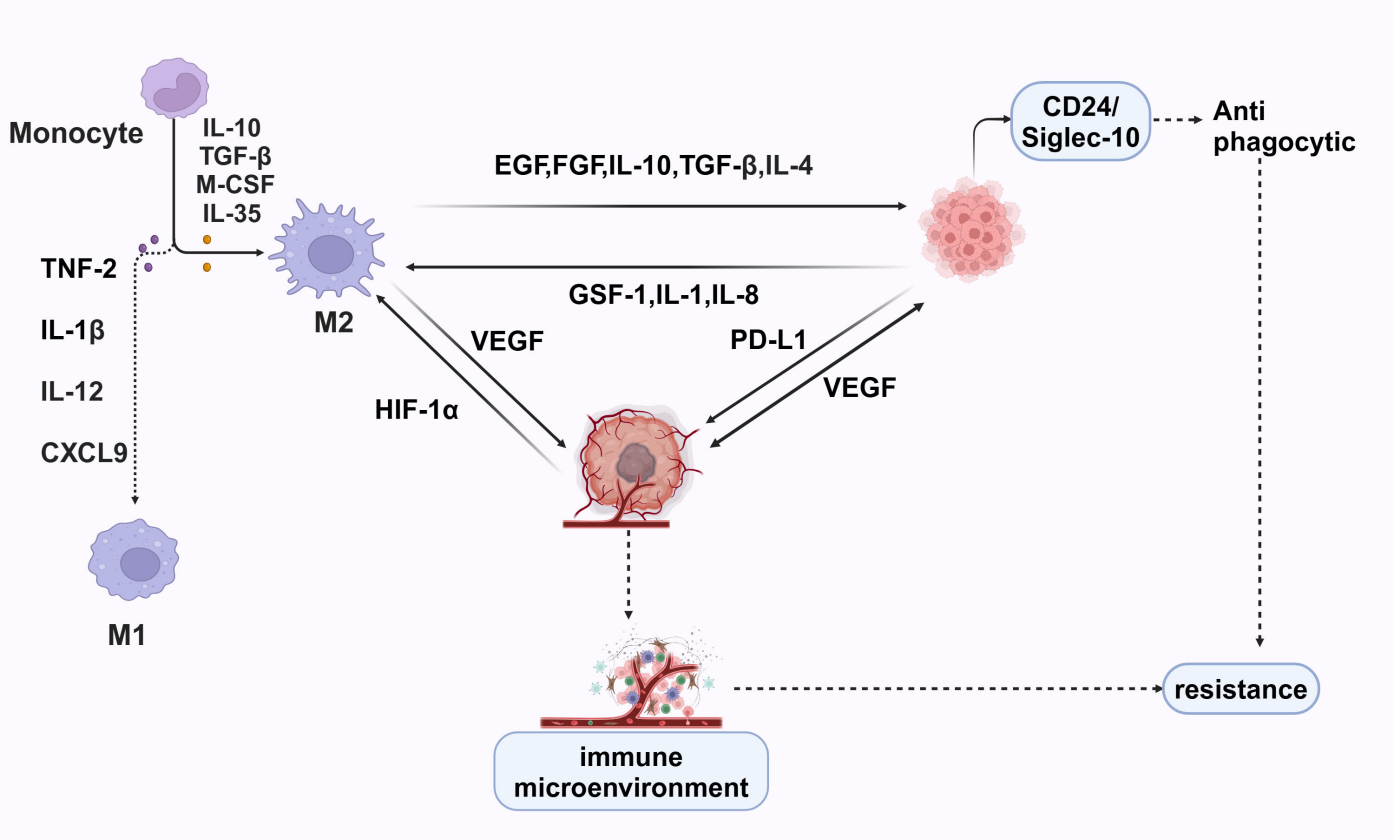
M1 macrophages, induced by TNF-2, IL-1β, IL-12, and CXCL9, have pro-inflammatory and anti-tumor functions, enhancing immune responses. In contrast, M2 macrophages, driven by IL-10, TGF-β, M-CSF, and IL-35, support tissue repair and tumor progression by secreting EGF, FGF, IL-10, TGF-β, IL-4, VEGF, and PD-L1, promoting tumor growth and immune suppression. Hypoxia-inducible factor 1-alpha (HIF-1α) exacerbates this by upregulating VEGF. Cancer cells further attract M2 macrophages via GSF-1, IL-1, and IL-8, creating a pro-tumorigenic environment. The CD24/Siglec-10 interaction provides anti-phagocytic signals, helping cancer cells evade destruction.

Macrophages polarize into M1 macrophages under the influence of cytokines such as IFN-γ and TNF-α. M1 macrophages are characterized by high expression of CD86 and CD80. They secrete cytokines and chemokines, including TNF-α, IL-1β, IL-12, and CXCL10, which promote the pro-inflammatory Th1 response (103). However, under certain conditions, M1 macrophages can exacerbate inflammation, potentially harming health. They are also capable of phagocytosing a significant number of pathogens and eliminating intracellular bacteria (116). In the presence of IL-4, TGF-β, IL-10, M-CSF, and IL-13, macrophages polarize into M2 macrophages. M2 macrophages are essential for normal immune functions, such as stimulating the Th2 response, regulating immunity, and facilitating tissue regeneration. However, certain subsets of M2 macrophages also significantly promote tumor progression (117) (**Figure 5**).

Tumor cells can evade immune surveillance and develop resistance due to the immunosuppressive microenvironment surrounding them. Clinically, this is evident as only a minority of cancer patients exhibit sustained responses when treated with immune checkpoint inhibitors or related drugs, while most patients do not derive substantial benefits from monotherapy targeting PD-1/PD-L1. Beyond the primary resistance of tumors to anti-PD-1/PD-L1 therapy, a significant subset of responders develops acquired resistance after the initial response (118). The primary mechanisms underlying tumor resistance encompass various factors, such as the presence of an immunosuppressive microenvironment, immunosuppressive factors, over expression of VEGF, T cell dysfunction, and other resistance patterns.

The development of tumor resistance is closely related to the surrounding immune microenvironment. A key issue is how to regulate and promote the formation of an immune-supportive environment.

### 5.1 Restore the phagocytosis of macrophages

M2-type tumor-associated macrophages (TAMs) play a significant role in the tumor microenvironment by suppressing inflammation, promoting tumor growth, and facilitating angiogenesis. Consequently, they contribute to tumor development. Transforming M2-type macrophages into M1-type macrophages within the tumor is considered a potential strategy to improve the tumor microenvironment and enhance the anti-tumor immune response (119, 120).

Regarding drug therapy, specific drugs have been identified that inhibit signaling pathways in M2-type macrophages, leading to their transformation into M1-type macrophages. Examples include certain non-steroidal anti-inflammatory drugs (NSAIDs), signal transduction inhibitors, and other agents. Additionally, certain cytokines, such as interferon and TNF, have shown the ability to promote the conversion of M2-type macrophages into M1-type macrophages (121–123).

**Figure 6:**
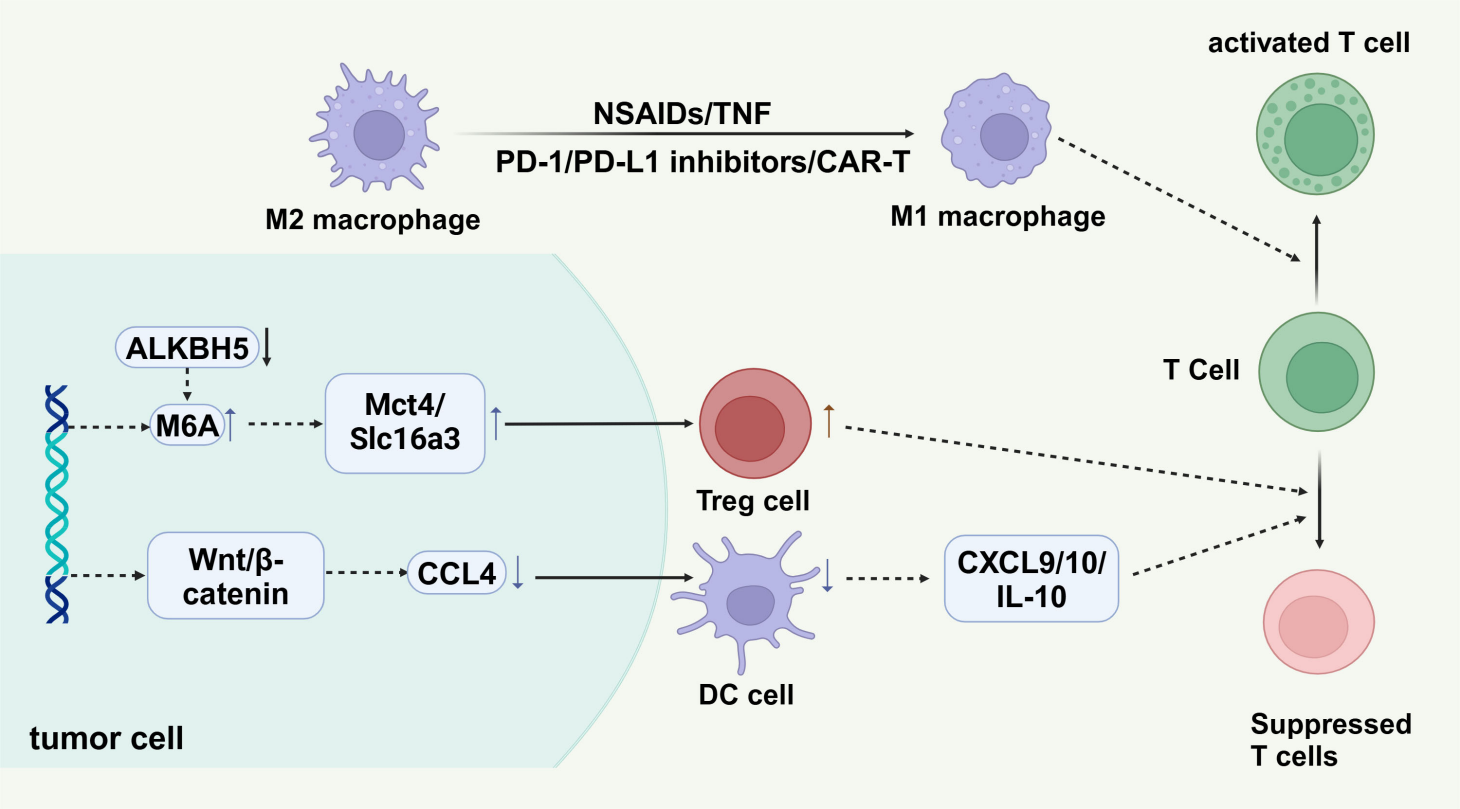
Tumor cells create an immunosuppressive environment through mechanisms such as the ALKBH5 enzyme, which regulates m6A RNA modification, influencing the expression of metabolic transporters Mct4 and Slc16a3. These transporters aid in recruiting regulatory T cells (Treg cells), promoting tumor immune evasion. The Wnt/β-catenin pathway in tumor cells upregulates chemokine CCL4, attracting dendritic cells (DC cells) that secrete cytokines (CXCL9, CXCL10, IL-10), suppressing T cell activity. M2 macrophages support immunosuppression, while M1 macrophages, activated by agents like NSAIDs or CAR-T cell therapy, enhance T cell activation, crucial for anti-tumor responses.

Immunotherapy has also gained significant attention for its potential in promoting the transformation of M2-type macrophages into M1-type. Approaches such as immune checkpoint inhibitors (PD-1/PD-L1 inhibitors), CAR-T cell therapy, and other methods can stimulate the body’s immune system, thereby influencing the polarization state of tumor-associated macrophages (124, 125) (**Figure 6**).

It should be noted that the majority of these methods are still in the laboratory research stage, and further clinical studies and validation are necessary before their application in a clinical setting. Additionally, it is crucial to consider the specific type of tumor and individualized features when selecting and applying these methods for precision treatment.

### 5.2 Regulating hypoxic environment

The hypoxic microenvironment within tumors fosters the accumulation of hypoxia-inducible factor-1α (HIF1α). This factor leverages the substantial lactic acid produced by tumor cells via the “Warburg effect” to induce the expression of VEGF and promote the M2-like polarization of tumor-associated macrophages (TAMs) (126, 127). Studies have reported that ALKBH5 plays a crucial role in regulating the immune response within the tumor microenvironment by controlling the levels of lactic acid and inhibitory immune cell accumulation (128). M6A RNA modification plays a critical role in tumor initiation and progression. Deletion of the M6A demethylase Alkbh5 has been shown to improve the tumor microenvironment. Upon knockout of ALKBH5 in tumor cells, the Mct4/Slc16a3 pathway regulates lactic acid concentration and the accumulation of myeloid-derived suppressor cells (MDSCs) and regulatory T cells (Tregs) within the tumor. Consequently, the overall lactic acid content in the tumor microenvironment decreases, along with reduced levels of VEGF. This context has also been associated with improved effectiveness of anti-PD-1 therapy (128, 129).

Furthermore, HIF1α can stimulate the expression of CD39, CD73, and adenosine receptors on the surface of macrophages. CD73 breaks down AMP into adenosine, which subsequently acts on adenosine receptors to trigger M2-like polarization of TAMs (130, 131).

The hypoxic environment within tumors promotes the stability of the HIF-1 transcription complex by inhibiting prolyl hydroxylase (PHDs) activity, which prevents HIF-1α from being degraded by VHL-mediated proteasomal degradation. This process plays a crucial role in tumor growth and progression because HIF-1α is an important transcription factor that regulates the adaptation of tumor cells to low-oxygen conditions. Studies have shown that PHDs mark specific proline residues of HIF-1α for VHL-mediated degradation through hydroxylation. Under hypoxic conditions, PHD activity is inhibited, leading to increased stability of HIF-1α (132, 133).

In addition, the inhibition of PHDs is not only achieved through hypoxic conditions but can also be accomplished through pharmacological interventions. For example, studies have found that certain small molecule inhibitors can effectively suppress the activity of PHDs, thereby increasing the stability and activity of HIF-1α. These inhibitors show potential application value in the treatment of hypoxic diseases (134, 135).

Therefore, targeting HIF or adenosine represents a promising strategy for reprogramming the immunosuppressive tumor microenvironment.

### 5.3 Targeting cytokines and chemokines

The Wnt/β-catenin signaling pathway is a highly conserved pathway that plays a pivotal role in regulating various cellular processes, including differentiation, apoptosis, and wound healing (136). Additionally, this pathway is significantly involved in tumor immunity. Activation of the Wnt/β-catenin signaling pathway in cancer cells can lead to a reduction in dendritic cell (DC) recruitment by down-regulating the expression of CCL4. Consequently, this down-regulation limits the secretion of CXCL9/10 and IL-10, restraining the infiltration and activation of CD8+ T cells (**Figure 6**). Moreover, Wnt/β-catenin activity promotes the differentiation of CD4+ T cells into TH1 and TH2 cells, contributing to immunosuppression. Given the fundamental role of Wnt/β-catenin signaling in normal cells, it may be more therapeutically viable to target downstream modulators of the pathway to avoid potential toxicity associated with direct targeting. This approach would encompass regulating the tumor microenvironment (TME), targeting Treg and MDSC cells, as well as activating, initiating, and infiltrating T cells (137).

In a model representing the lack of response to immune checkpoint inhibitors (ICI) in non-alcoholic steatohepatitis-associated hepatocellular carcinoma (NASH-HCC), the combined administration of a CXCR2 antagonist and anti-PD1 has demonstrated efficacy in reducing tumor burden and extending survival. This combination therapy is associated with heightened activation of dendritic cells and an increased presence of CD8+ T cells, both linked to anti-tumor immunity. Consequently, CXCR2 inhibitors have the potential to reprogram the NASH-HCC tumor immune microenvironment, fostering a more favorable response to immune checkpoint inhibitors (138).

The cytokine Oncostatin M (OSM) contributes to the advancement of breast cancer by modifying the tumor microenvironment. Specifically, OSM can reprogram fibroblasts of myeloid origin, causing them to adopt a more contractile and tumorigenic phenotype. This reprogramming process is accompanied by the release of vascular endothelial growth factor (VEGF) and pro-inflammatory chemokines such as CXCL1 and CXCL16. These factors play a crucial role in promoting the recruitment of myeloid cells, facilitating the progression of breast cancer. Consequently, OSM has been identified as a promising therapeutic target for treating tumors (139).

## 6 Combination of immune checkpoint inhibitors and anti-angiogenic strategies

### 6.1 The related mechanism of combined therapy

Antiangiogenic therapy has the potential to enhance anti-tumor immunity by inhibiting multiple immunosuppressive characteristics of angiogenesis. A comprehensive understanding of how tumors evade immunity and the mechanisms underlying resistance to immunotherapy can facilitate the development of effective combination therapies (140).

Several studies have indicated that the administration of angiogenic inhibitors alone does not result in significant survival benefits for patients. The efficacy of angiogenesis inhibitors is often short-term and limited to specific tumor types. Furthermore, long-term use of these inhibitors may even promote tumor invasion, as seen in cases of pancreatic cancer and glioblastoma (38, 141). This suggests that the formation of an immunosuppressive microenvironment is mediated by multiple signaling pathways (38).

The administration of anti-angiogenic drugs may contribute to the anti-tumor immune response due to the association between certain pro-angiogenic molecules and immunosuppression. By blocking the signaling pathways involved in angiogenesis, it is possible to effectively reshape the immune-supportive microenvironment and enhance the effectiveness of immune checkpoint inhibitors (142). Combination therapy can regulate the expression of PD-L1 on dendritic cells, leading to improved T-cell function and increased T-cell numbers (143). Following combined therapy, the tumor microenvironment transforms into an immune-supportive microenvironment, characterized by an increase in cytotoxic T lymphocytes (CTLs), M1-like macrophages, and mature dendritic cells (DCs), along with a decrease in regulatory T cells (Tregs) (144). IFN-γ plays a crucial role in cell inhibition, apoptosis promotion, and immune stimulation, making it an important cytokine in anti-tumor immunity. Combination therapy can facilitate the activation of IFN-γ, promoting vascular normalization and regression (145). It has been shown to enhance the infiltration of effector cells such as natural killer (NK) cells and DCs into tumors while reducing the proliferation of immunosuppressive cells, including Tregs and myeloid-derived suppressor cells (MDSCs). Additionally, combination therapy leads to the polarization of tumor-associated macrophages (TAMs) towards an immune-supporting M1 phenotype (146).

In summary, following combined therapy, the increase in anti-tumor factors and the decrease in tumor-promoting factors alleviate the immunosuppressive tumor microenvironment, thereby improving the efficacy of tumor immunotherapy.

### 6.2 Clinical trials investigating the efficacy of ICIs combined with anti-angiogenic therapy

Immune checkpoint inhibitors (ICIs) combined with anti-angiogenic agents exhibit synergistic effects, providing significant therapeutic advantages in various solid tumors. This is strongly supported by existing clinical trial evidence. For example, the APICAL-RST trial is a Phase II study that evaluates the efficacy and safety of combining anlotinib with PD-1 inhibitors in patients with heavily pre-treated, refractory metastatic solid tumors. In this open-label trial, patients who experienced disease progression during prior treatments received the combination therapy of anlotinib and PD-1 inhibitors. The results indicated that the objective response rate (ORR) in the intention-to-treat population was 22.0%, while the disease control rate (DCR) was 73.2%. Among patients with evaluable efficacy, the ORR reached 81.1%. Furthermore, 63.4% of patients had a PFS2/PFS1 ratio greater than 1.3, suggesting an improvement in progression-free survival. Overall, this study demonstrates that this combination therapy is effective and well-tolerated in patients with refractory solid tumors (147) (**Table 1**, https://clinicaltrials.gov).

**Table 1:** Clinical trials investigating the efficacy of ICIs combined with anti-angiogenic therapy.

Cancer	Trials ID	Phase	Treatment	N
NPC	NCT04586088	II	Camrelizumab and Apatinib	58
Pan cancer	NCT03239015	II	PD-1 inhibitor and Anlotinib	41
TNBC	NCT03394287	II	Camrelizumab and Apatinib	40
NSCLC	NCT03628521	Ib	Sintilimab and Anlotinib	22
EC	NCT03517449	III	Pembrolizumab and Lenvatinib	411
RCC	NCT03141177	III	Nivolumab and Cabozantinib	323
BEATcc	NCT03556839	III	Atezolizumab and Bevacizumab	410
HCC	NCT03434379	III	Atezolizumab and Bevacizumab	336
CRC	NCT04715633	II	Camrelizumab and Apatinib	53

NPC, nasopharyngeal carcinoma; NSCLC, non-small cell lung cancer; TNBC, triple-negative breast cancer; EC, Endometrial Cancer; RCC, Renal-Cell Carcinoma; BEATcc, recurrent cervical cancer; HCC, Hepatocellular Carcinoma; CRC, Colorectal Cancer.

A study investigated the efficacy and safety of combining camrelizumab and apatinib in patients with recurrent or metastatic nasopharyngeal carcinoma (NPC) who had shown no response to at least one line of systemic therapy. Patients received 200 mg of camrelizumab every three weeks and 250 mg of apatinib daily. The objective response rate was 65.5%, and the disease control rate was 86.2%. The median progression-free survival was 10.4 months. However, 58.6% of patients experienced moderate to severe treatment-related adverse events, with common issues including hypertension and nasopharyngeal necrosis. The results indicate that this combination therapy demonstrates good anti-tumor activity, although there are associated safety concerns (148).

In a phase II trial, patients with advanced triple-negative breast cancer (TNBC) were treated with a combination of Camrelizumab and Apatinib. The treatment regimen involved administering Camrelizumab intravenously every two weeks alongside Apatinib, which was given either continuously (from day 1 to day 14) or intermittently (from day 1 to day 7). The objective response rate (ORR) for the continuous dosing cohort was 43.3%, with 13 out of 30 patients showing an objective response, while the intermittent dosing cohort showed no objective responses. The disease control rate was 63.3% in the continuous dosing cohort and 40.0% in the intermittent cohort. The median progression-free survival (PFS) was 3.7 months for the continuous dosing group and 1.9 months for the intermittent group. In conclusion, the combination of Camrelizumab and Apatinib provided a significantly higher ORR than previously reported with either anti-PD-1/PD-L1 antibodies or Apatinib monotherapy, demonstrating promising therapeutic effects and a manageable safety profile in advanced TNBC patients (149).

A study evaluated the efficacy and safety of sintilimab combined with anlotinib as a first-line treatment for advanced non-small cell lung cancer (NSCLC) in treatment-naive patients. In this phase 1b trial, eligible patients with unresectable stage IIIB/C or IV NSCLC without EGFR/ALK/ROS1 mutations received sintilimab at a dose of 200 mg on day 1 and anlotinib at a dose of 12 mg from day 1 to day 14, administered every three weeks until disease progression or unacceptable toxicity occurred. The objective response rate (ORR) was reported at 72.7%, with 16 out of 22 patients achieving confirmed partial responses. The study also found a disease control rate of 100% and a median progression-free survival (PFS) of 15 months. In conclusion, this trial represents the first assessment of an anti-PD-1 antibody combined with a multitarget antiangiogenic tyrosine kinase inhibitor in a frontline setting for NSCLC patients. Given the promising efficacy, durability, and safety profile, the combination of sintilimab and anlotinib offers a novel chemotherapy-free treatment option for this patient population (150).

Additionally, combinations such as the anti-PD-1 antibody carlizumab with apatinib for treating cT4a/bN+ gastric cancer (151), immune checkpoint inhibitors (ICIs) combined with anti-angiogenic therapy and radiotherapy (RT) for hepatocellular carcinoma (HCC) (152), a novel humanized IgG1 antibody targeting PD-L1 combined with anlotinib for triple-negative breast cancer (153), and anti-PD-1 antibodies combined with vascular kinase inhibitors targeting the tumor microenvironment in malignant mesothelioma (154) have all demonstrated promising antitumor effects. Additional trials are currently in the recruitment phase (**Table 2**, https://clinicaltrials.gov).

**Table 2:** Some ongoing clinical trials that are currently recruiting participants.

Cancer	Trials ID	Treatment	Status
HCC	NCT06117891	Atezolizumab and Bevacizumab	Recruiting
HCC	NCT06096779	Atezolizumab and Bevacizumab	Recruiting
MUM	NCT05308901	Pembrolizumab and Lenvatinib	Recruiting
Sarcomas	NCT05182164	Pembrolizumab and Cabozantinib	Recruiting
RCC	NCT03341845	Avelumab and Axitinib	Recruiting

HCC, Hepatocellular Carcinoma; MUM, Metastatic Uveal Melanoma; RCC, Renal-Cell Carcinoma.

However, while combination therapy can be effective in treating tumors, it is not without potential risks. For instance, combining immune checkpoint inhibitors with angiogenesis inhibitors can increase the risk of cardiovascular toxicity (155). The therapeutic effect of immune checkpoint inhibitors (ICI) has demonstrated variability based on sex. Notably, ICI therapy has shown significant survival benefits for patients with melanoma. However, there are gender disparities in the response to ICI treatment, with men deriving greater benefits compared to women. Despite this observed difference, the underlying mechanism remains unclear. A study has revealed that the combination of immune checkpoint inhibitors (ICI) and bevacizumab independently increases the risk of interstitial lung disease, hypertension, and gastrointestinal bleeding. Additionally, when considering combination therapy, it is crucial to carefully evaluate the potential for endocrine-related adverse reactions (156). These findings emphasize the importance of closely monitoring patients receiving ICI and bevacizumab co-treatment and taking appropriate precautions to reduce the risks associated with these adverse events.

### 6.3 Application of ICIs combined with anti-angiogenesis in hepatocellular carcinoma: a positive case

Hepatocellular carcinoma (HCC) is one of the most common malignancies globally, accounting for approximately 6% of all human cancers, with a mortality rate of 8.3%, making it the third leading cause of cancer-related deaths worldwide (157). In 2020, over 900,000 new cases were reported, and more than 830,000 deaths occurred. The overall survival time for HCC patients is extremely short, with a 5-year survival rate of less than 10% (158). The tumor microenvironment (TME) in HCC is complex, characterized by high vascularization and an immunosuppressive state. Angiogenesis is crucial for tumor growth, invasion, and metastasis, as tumors rely on the formation of new blood vessels to supply nutrients and oxygen. Simultaneously, the immunosuppressive TME in HCC enables tumor cells to evade immune surveillance and attack (159).

Immune checkpoint inhibitors (ICIs) and anti-angiogenesis agents have emerged as two key classes of drugs in cancer therapy. The combined use of these therapies, targeting different aspects of the TME, has shown immense potential in improving the prognosis of HCC patients.

The IMBrave-150 trial marked a milestone in HCC treatment, establishing the combination of atezolizumab and bevacizumab as the new standard of care for advanced HCC. This regimen demonstrated a significant overall survival (OS) benefit, with a median OS of 19.2 months, compared to 13.2 months with sorafenib. Additionally, progression-free survival (PFS) was substantially improved, with a median PFS of 6.9 months versus 4.3 months for sorafenib. This combination also led to a significantly higher objective response rate (ORR) of 30%, in contrast to 5% with sorafenib, highlighting the potential of combining immune checkpoint inhibition with anti-angiogenesis therapy in this setting (160).

Another notable trial, the HIMALAYA study, evaluated the combination of durvalumab and a single priming dose of tremelimumab in patients with advanced HCC. The combination therapy significantly improved OS compared to sorafenib, with a median OS of 16.4 months versus 13.8 months. Furthermore, the combination achieved an ORR of 20.1%, compared to 5.1% for sorafenib, and demonstrated a longer duration of response (DOR) of nearly two years, emphasizing the sustained anti-tumor activity of this combination (161).

In the CheckMate-040 trial, the combination of nivolumab and ipilimumab was assessed in patients with advanced HCC who had previously been treated with sorafenib. This combination showed promising results, with an ORR of 32%, a median DOR of 17.5 months, and an OS of 22.8 months after a minimum follow-up of 44 months, illustrating its potential as a viable treatment option for patients previously treated with sorafenib (162).

The KEYNOTE-524 Phase Ib trial evaluated the combination of lenvatinib and pembrolizumab in patients with unresectable HCC. The study demonstrated an impressive ORR of 46.0% and 36.0%, according to mRECIST and RECIST v1.1 criteria, respectively. The median durations of response were 8.6 months and 12.6 months, while the median PFS was 9.3 and 8.6 months. Importantly, the median OS was 22 months, indicating the potential of this combination as a first-line treatment option (163).

The LEAP-002 Phase III study compared the efficacy and safety of pembrolizumab in combination with lenvatinib versus lenvatinib alone as a first-line treatment for advanced HCC. Although the combination did not significantly improve OS or PFS, it resulted in the longest OS observed to date for first-line treatment in HCC, with a median OS of 21.2 months, underscoring the promising potential of combining immune checkpoint inhibitors with anti-angiogenesis therapies (164).

A Phase II single-arm trial investigated the combination of donafenib and sintilimab as a first-line treatment for advanced HCC. This combination demonstrated promising anti-tumor activity and an acceptable safety profile, suggesting its potential for broader clinical application in HCC treatment (165).

## 7 Biomarkers

Can we extract certain biomarkers from patients to assess their condition and disease status, thereby selecting the most appropriate treatment? Unfortunately, there are currently few reports on this topic. This article reviews several biomarkers, with the hope of contributing to improved treatment strategies for the disease.

A study explored the effectiveness of combining immune checkpoint inhibitors (ICIs) with anti-angiogenesis therapy in patients with esophageal cancer, focusing on three angiogenesis-related biomarkers: IL-8, TIE2, and HGF. The baseline levels of these biomarkers were significantly associated with patient survival outcomes, effectively predicting responses to immunotherapy and prognosis. Additionally, the research found that the combination of anti-angiogenesis therapy with ICIs significantly improved overall survival compared to the use of ICIs alone. This indicates that angiogenesis-related biomarkers play an important role in optimizing immunotherapy and enhancing treatment efficacy in esophageal cancer (166).

A study examines the role of tertiary lymphoid structures (TLS) as a potential biomarker for predicting outcomes in patients with combined hepatocellular-cholangiocarcinoma (cHCC-CCA) receiving immune checkpoint inhibitors and anti-angiogenic therapies. A high intratumoral TLS score is linked to longer survival, while a high TLS density in surrounding tissues suggests worse prognosis. Mature TLSs are associated with better outcomes and greater infiltration of CD8+ T cells.The researchers also classified patients into four immune grades based on TLS distribution, which were identified as independent prognostic factors. Additionally, TLS presence correlated with CXCL12 expression in cHCC-CCA tissues, highlighting its significance in the tumor’s immune microenvironment. Overall, the distribution and density of TLSs are crucial for understanding the immune environment in cHCC-CCA, affecting prognosis and serving as potential biomarkers for immunotherapy response (167, 168).

In a Phase II trial, 118 patients with advanced hepatocellular carcinoma (HCC) were treated with camrelizumab and apatinib. The study measured blood-based maximum somatic allele frequency (bMSAF) in peripheral blood samples, revealing a correlation between bMSAF and vascular invasion as well as alpha-fetoprotein (AFP) levels. Notably, lower bMSAF values were significantly associated with extended progression-free survival (PFS) and overall survival (OS). This observation suggests that higher bMSAF levels may indicate a greater tumor burden, leading to immune response suppression. In patients undergoing first-line treatment, the impact of bMSAF on survival and response rates was not statistically significant. However, it is important to note that the association between bMSAF and PFS approached significance. Overall, in patients with advanced HCC receiving the combination of camrelizumab and apatinib, bMSAF emerged as a more valuable baseline circulating biomarker than blood tumor mutational burden (bTMB). Furthermore, bMSAF serves as a prognostic predictor for patients treated with the immune checkpoint inhibitor camrelizumab and the anti-angiogenic agent apatinib (169).

There is increasing evidence that peripheral blood biomarkers, such as exosomes, circulating tumor DNA, and peripheral blood proteins, play significant roles in cellular physiological functions (170, 171). Furthermore, extracellular vesicles (EVs) serve as crucial mediators of intercellular communication in cancer, significantly influencing the regulation of the tumor immune microenvironment and facilitating immune evasion. Emerging evidence indicates that EV-derived non-coding RNAs not only modulate the biological functions of tumor cells but also play a pivotal role in regulating immune cell functions and reprogramming macrophages (172, 173). Consequently, integrating these therapeutic approaches with EV-mediated immune modulation offers promising potential for advancing the treatment of malignant cancers, including lung cancer. They can provide extensive information representing the tumor immune microenvironment. In conclusion, we hope to develop more biomarkers that reflect the conditions within the patient’s body, assisting patients in selecting better treatment options.

## 8 Conclusion

This review examines the pivotal roles of immune checkpoint inhibitors (ICIs) and anti-angiogenic therapies in enhancing anti-tumor immunity. It highlights their potential to improve disease control rates and patient survival, positioning these strategies as promising advancements in cancer treatment. Furthermore, approaches targeting macrophage reprogramming have demonstrated efficacy in restoring their anti-tumor functions. The review also underscores the importance of identifying novel biomarkers to facilitate personalized treatment strategies. Moving forward, further research is needed to elucidate the underlying mechanisms of these therapies and to promote their clinical application, ultimately aiming to improve treatment outcomes and quality of life for cancer patients.

## Glossary

4-1BB: 4-1BB co-stimulatory molecule
ATF6: Activating transcription factor 6
bMSAF: Blood-based maximum somatic allele frequency
bTMB: Blood tumor mutational burden
CAR-T: Chimeric antigen receptor T cell
CD47: Cluster of differentiation 47
CTLA-4: Cytotoxic T-lymphocyte-associated protein 4
CSF-1: Colony-stimulating factor 1
CXCL1: C-X-C motif chemokine ligand 1
CXCL16: C-X-C motif chemokine ligand 16
CXCR2: C-X-C chemokine receptor type 2
DCs: Dendritic cells
DCR: Disease control rate
EVs: Extracellular vesicles
ER: Endoplasmic reticulum
HEVs: High endothelial venules
HIF-1α: Hypoxia-inducible factor-1 alpha
ICIs: Immune checkpoint inhibitors
ICOS: Inducible T-cell costimulator
IL-6: Interleukin-6
IL-10: Interleukin-10
IRE1: Inositol-requiring enzyme 1
LAG-3: Lymphocyte activation gene 3
LTβR: Lymphotoxin beta receptor
MDSCs: Myeloid-derived suppressor cells
MSI: Microsatellite instability
NK cells: Natural killer cells
OS: Overall survival
ORR: Objective response rate
OSM: Oncostatin M
PD-1: Programmed cell death protein 1
PD-L1: Programmed death-ligand 1
PERK: PRKR-like endoplasmic reticulum kinase
PFS: Progression-free survival
SIRPα: Signal regulatory protein alpha
TAMs: Tumor-associated macrophages
TGF-β: Transforming growth factor-beta
TIGIT: T cell immunoglobulin and immunoreceptor tyrosine-based inhibitory motif domain
TIM-3: T cell immunoglobulin and mucin domain-containing protein 3
TNBC: Triple-negative breast cancer
TME: Tumor microenvironment
Tregs: Regulatory T cells
UPR: Unfolded protein response
VEGF: Vascular endothelial growth factor
VEGFR2: Vascular endothelial growth factor receptor 2
